# Multi-Dimensional Anomaly Detection and Fault Localization in Microservice Architectures: A Dual-Channel Deep Learning Approach with Causal Inference for Intelligent Sensing

**DOI:** 10.3390/s25113396

**Published:** 2025-05-28

**Authors:** Suchuan Xing, Yihan Wang, Wenhe Liu

**Affiliations:** 1 Department of Electrical and Computer Engineering, Duke University, Durham, NC 27708, USA; sx80@alumni.duke.edu; 2 School of Engineering and Applied Science, The University of Pennsylvania, Philadelphia, PA 19104, USA; 3 School of Computer Science, Carnegie Mellon University, Pittsburgh, PA 15213, USA

**Keywords:** anomaly detection, fault localization, microservice architecture, monitoring system, performance sensing

## Abstract

Modern data centers face increasing complexity with distributed microservice architectures, making anomaly detection and fault localization challenging yet critical. Traditional monitoring sensor tools struggle with heterogeneous metrics, temporal correlations, and precise root cause analysis in these environments. This paper proposes a dual-channel deep learning framework that integrates Temporal Convolutional Networks with Variational Autoencoders to address these challenges. Our approach employs contrastive learning to create unified representations of diverse service metrics and incorporates causal inference mechanisms to trace fault propagation paths. We evaluated our framework using a semi-supervised learning approach that leveraged both labeled anomalies and abundant normal data, achieving 95.4% detection accuracy, 93.8% F1-score, and 87.6% precision in fault component localization. The system reduced the average troubleshooting time by 43% and false localization rates by 31% compared to state-of-the-art methods, while maintaining a computational efficiency suitable for real-time monitoring. These results demonstrate the effectiveness of our approach in identifying and precisely localizing anomalies in complex microservice environments through intelligent sensing of system metrics, enabling proactive maintenance strategies that minimize service disruptions.

## 1. Introduction

Modern cloud computing infrastructures are increasingly built on complex microservice architectures, where applications are composed of numerous loosely coupled services that communicate through well-defined interfaces [1]. While this architectural paradigm offers significant advantages in terms of scalability, flexibility, and development efficiency, it also introduces substantial challenges for system monitoring, anomaly detection, and fault diagnosis [2]. The distributed nature of these systems, combined with their dynamic scaling capabilities and intricate service dependencies, creates an environment where the performance issues captured by monitoring sensors can rapidly propagate across the infrastructure, making it difficult to locate the root cause of problems [3]. The complexity of microservice environments is further amplified by the heterogeneous nature of monitoring data. System administrators must track numerous Key Performance Indicators (KPIs) across multiple dimensions, including CPU utilization, memory consumption, network traffic, response times, error rates, and application-specific metrics sensed from distributed service components [4]. These metrics often exhibit different statistical properties, temporal patterns, and correlation structures, creating significant challenges for traditional anomaly detection approaches [5].

Early detection and precise localization of anomalies through intelligent sensing are critical for maintaining service reliability and minimizing downtime in production environments. According to recent industry reports, the average cost of downtime for enterprise-level services exceeds USD 5600 per minute, with potential financial impacts reaching millions of dollars per hour for major online service providers. Despite these high stakes, existing monitoring solutions frequently struggle with false positives, delayed detection, and imprecise fault localization [6]. Traditional threshold-based methods for anomaly detection lack the sophistication required to handle the dynamic nature of microservice metrics and often fail to capture complex temporal dependencies and inter-service correlations [7]. More advanced machine learning approaches, including isolation forests [8], one-class SVMs [9], and clustering-based methods [10], have demonstrated improved capabilities but typically treat metrics in isolation, overlooking the multi-dimensional relationships that characterize microservice failures.

Recent advancements in deep learning have shown promise for addressing these limitations. Recurrent Neural Networks (RNNs) and Long Short-Term Memory (LSTM) networks have been applied to capture temporal dependencies in monitoring data [11], while autoencoders have proven effective for learning compact representations of normal system behavior [12]. However, most existing deep learning approaches primarily focus on anomaly detection, without addressing the equally important challenge of fault localization—identifying which specific component or service is responsible for an observed anomaly [13]. Furthermore, the causal relationships between metrics and the propagation patterns of failures across services remain largely unexplored in current research. Understanding these causal mechanisms is essential for accurate root cause analysis and effective remediation strategies [14]. The lack of integrated approaches that combine anomaly detection with causal analysis presents a significant gap in the literature and limits the practical utility of existing solutions [15].

To address these challenges, we propose a novel dual-channel deep learning framework that combines Temporal Convolutional Networks (TCNs) with Variational Autoencoders (VAEs) for joint anomaly detection and fault localization in microservice architectures. Our approach leverages contrastive learning to create unified representations of heterogeneous metrics and incorporates causal inference mechanisms to trace fault propagation paths across services. By integrating these components, our framework not only identifies anomalies with high accuracy but also pinpoints their root causes, enabling more efficient troubleshooting and maintenance processes. It is important to clarify that our framework follows a semi-supervised learning paradigm, utilizing both abundant normal operational data and limited labeled anomaly examples from historical incidents during training. While the majority of anomaly detection research focuses on unsupervised approaches that rely solely on normal data patterns, the semi-supervised paradigm is particularly relevant for production microservice environments, where some historical anomaly incidents with known root causes are typically available and the high cost of critical failures justifies leveraging this supervisory signal to improve detection accuracy and reduce false positives. The main contributions of this paper can be summarized as follows:

- We design a dual-channel architecture that combines the temporal modeling capabilities of TCNs with the latent representation power of VAEs, enabling effective anomaly detection across diverse metrics and service types.
- We develop a contrastive learning approach that creates unified representations of heterogeneous monitoring data, addressing the challenge of correlating metrics with different statistical properties and scales.
- We propose a causal inference mechanism that models the dependencies between services and traces fault propagation patterns, significantly improving the precision of root cause localization.
- We conducted extensive experiments on the public MultiDimension-Localization dataset, demonstrating that our framework outperformed state-of-the-art methods in both anomaly detection and fault localization tasks.
- We analyzed the computational efficiency and scalability of our approach, showing that it maintains real-time performance, even with large numbers of services and metrics.

The remainder of this paper is organized as follows: Section 2 reviews related work on anomaly detection, fault localization, and causal inference. Section 3 presents the problem formulation and details our proposed methodology. Section 4 describes the experimental setup, dataset characteristics, and evaluation metrics. Section 5 discusses the experimental results and compares our approach with existing methods. Section 6 concludes our work and outlines directions for future research. concludes the paper.

## 2. Related Works

This section reviews the existing literature related to anomaly detection and fault localization in distributed systems, with a focus on microservice architectures. We categorize related work into three main areas: (1) traditional anomaly detection methods, (2) deep learning approaches for anomaly detection, and (3) fault localization and root cause analysis techniques.

### 2.1. Traditional Anomaly Detection Methods

Traditional anomaly detection approaches in distributed systems typically rely on statistical methods, machine learning algorithms, or rule-based techniques. Statistical methods include threshold-based detection [16], moving average processes [17], and time series decomposition [18]. These approaches generally assume that normal behavior follows certain statistical distributions, and they then flag deviations from these sensed patterns as anomalies. Machine-learning-based approaches have gained significant attention due to their ability to learn complex patterns without explicit programming. Supervised methods require labeled anomaly data for training, which are often scarce in real-world scenarios [19]. In contrast, unsupervised methods such as clustering, isolation forests [8], and one-class SVM [9] can identify anomalies without labeled training data by detecting instances that deviate significantly from the majority. Rule-based systems employ domain knowledge to establish detection rules [20]. While effective in well-understood environments, these systems often struggle to adapt to the dynamic nature of microservice architectures, where service behaviors and interactions evolve rapidly [21]. Additionally, the high dimensionality and heterogeneity of monitoring data in microservice environments create significant challenges for traditional methods, which typically process each metric independently and fail to capture complex inter-service dependencies [22].

### 2.2. Deep Learning for Anomaly Detection

Deep learning approaches have demonstrated superior performance in anomaly detection for complex systems, due to their ability to learn hierarchical representations and capture intricate temporal patterns. Recurrent Neural Networks, particularly Long Short-Term Memory networks, have been widely applied to time series anomaly detection in various domains, including network traffic [23], cloud computing [24], and IoT systems [25]. Autoencoders represent another popular class of deep learning models for anomaly detection. By learning to reconstruct normal behavior and using reconstruction error as an anomaly indicator, these methods have shown effectiveness in various applications [26]. VAEs extend traditional autoencoders by learning probabilistic latent representations, providing better generalization capabilities for complex data distributions [27]. DONUT [12] applies VAEs for unsupervised anomaly detection in seasonal KPIs of web applications, addressing challenges such as missing data and seasonal patterns. Recent research has explored TCNs for time series modeling, offering advantages over RNN-based architectures through parallel processing, flexible receptive fields, and stable gradients [28]. OmniAnomaly [29] combines stochastic recurrent neural networks with planar normalizing flows for multivariate time series anomaly detection, achieving state-of-the-art performance on various datasets. Despite these advancements, most existing deep learning approaches primarily focus on anomaly detection, without addressing the equally important task of fault localization, particularly in the context of microservice architectures where anomalies can propagate across multiple services [6].

### 2.3. Fault Localization and Root Cause Analysis

Fault localization in distributed systems aims to identify the root cause of observed anomalies, a challenging task due to complex service dependencies and fault propagation patterns. Traditional approaches rely on expert knowledge and rule-based systems [30], which become increasingly impractical as system complexity grows. Graph-based methods have gained prominence for modeling service dependencies and tracing fault propagation. CloudRanger [31] constructs a service dependency graph and leverages statistical techniques to identify the root cause of performance anomalies. Similarly, MicroScope [4] builds causal graphs to pinpoint performance issues in microservice environments, analyzing request trace data to infer causal relationships between services. Recent advances have incorporated machine learning techniques for more automated fault localization. MicroRCA [7] employs a random forest classifier to identify the root cause component based on anomaly propagation patterns. MIDAS [32] proposes a multi-dimensional anomaly detection and localization framework that combines autoencoders with attention mechanisms to identify faulty services. Causal inference techniques have emerged as a promising direction for more precise root cause analysis. CausalRCA [33] applies causal discovery algorithms to infer causal relationships between metrics and services, enabling more accurate fault localization. ClearCausal [34] leverages structural equation models to capture causal dependencies between services and metrics, demonstrating improved performance in complex microservice environments. Despite these advancements, existing approaches often struggle with the heterogeneity of monitoring data and the dynamic nature of microservice architectures. Most methods either focus on anomaly detection without localization or employ separate models for each task, potentially missing important correlations between them. Additionally, few approaches explicitly model both temporal dependencies and causal relationships, which are crucial for understanding fault propagation in distributed systems [35].

## 3. Preliminaries

This section introduces key concepts, notations, and problem definitions to establish the foundation for our methodology.

### 3.1. Problem Formulation

Microservice architectures consist of multiple loosely coupled services that communicate with each other through well-defined interfaces. In this context, we formally define the anomaly detection and fault localization problems as follows: Consider a microservice system with *N* services, where each service generates *M* different types of performance metrics over time. Let $X=\{x_{1},x_{2},\ldots ,x_{N\times M}\}$ represent the set of all time series metrics, where each $x_{i}=\left\{x_{i}^{1},x_{i}^{2},\ldots ,x_{i}^{T}\right\}$ is a sequence of observations for the *i*-th metric over *T* timestamps. Given this multivariate time series data, we aim to address two interconnected tasks:

- **Anomaly Detection**: Identify timestamps *t* where the system exhibits anomalous behavior, represented by a binary indicator function $f_{a}\left(X,t\right)\in \left\{0,1\right\}$, where 1 indicates an anomaly.
- **Fault Localization**: For each detected anomaly at time *t*, identify the specific service(s) $S_{root}\subseteq \left\{1,2,\ldots ,N\right\}$ that represent the root cause of the anomaly through a function $f_{l}\left(X,t\right)=S_{root}$.

In the context of microservice architectures, we define **anomalies** as deviations from normal system behavior observable through monitoring metrics, such as sudden spikes in response time, memory usage exceeding normal ranges, or error rate increases. **Faults** represent the underlying root causes of these anomalies, such as memory leaks in specific services, network congestion between service components, or database connection pool exhaustion. Our framework addresses four primary categories consistent with the dataset: resource contention (CPU/memory exhaustion), network latency issues, application errors (exceptions, timeouts), and dependency failures (service unavailability, cascading failures).

### 3.2. Key Technical Concepts

#### 3.2.1. Temporal Convolutional Networks

Temporal Convolutional Networks are specialized convolutional architectures designed for sequence modeling. Unlike recurrent networks, TCNs employ causal convolutions that prevent information leakage from future time steps to past ones. A key feature of TCNs is the use of dilated convolutions, which allow the network to effectively capture long-range temporal dependencies by exponentially expanding the receptive field without increasing the computational cost [28]. Formally, a dilated causal convolution operation on a 1D sequence input *x* is defined as

(1) $$F\left(s\right)=\left(x{*}_{d}f\right)\left(s\right)=\sum_{i=0}^{k-1}f\left(i\right)\cdot x_{s-d\cdot i}$$

where *f* is a filter of size *k*, *d* is the dilation factor, and s−d·i ensures causality.

#### 3.2.2. Variational Autoencoders

Variational Autoencoders are generative models that learn a probabilistic mapping between the data space and a lower-dimensional latent space [36]. Unlike traditional autoencoders, VAEs model the latent space as a probability distribution, typically a multivariate Gaussian. The VAE consists of an encoder network that maps input *x* to the parameters of a latent distribution (mean μ and variance $\sigma^{2}$), and a decoder network that reconstructs the input from samples drawn from this distribution. The objective function combines reconstruction quality with a regularization term:

(2) $$L_{VAE}=E_{q(z|x)}\left[\log p\left(x|z\right)\right]-D_{KL}\left(q\left(z|x\right)||p\left(z\right)\right)$$

where *x* is the input data, *z* is the latent variable, q(z|x) is the encoder posterior distribution, p(x|z) is the decoder likelihood, p(z) is the prior distribution (typically standard Gaussian), and $D_{KL}$ denotes the Kullback–Leibler divergence. In practice, the encoder network outputs the mean μ and log-variance log $\sigma^{2}$ through two separate fully connected layers that map the input features to the latent dimension. The mean and variance parameters are learned during training through backpropagation, with the encoder automatically determining appropriate latent representations for the input data distribution.

#### 3.2.3. Causal Inference

Causal inference aims to identify cause–effect relationships between variables, going beyond mere statistical correlations [37]. In the context of distributed systems, causal relationships can be represented as a directed acyclic graph (DAG) G=(V,E), where nodes *V* represent components or metrics, and edges *E* represent causal dependencies. Several algorithms have been developed to infer causal structures from observational data, including constraint-based methods like the PC algorithm [38] and score-based methods like Greedy Equivalence Search (GES) [39]. These algorithms identify conditional independence relationships between variables to construct or score potential causal graphs.

#### 3.2.4. Contrastive Learning

Contrastive learning is a self-supervised learning technique that learns representations by maximizing the agreement between differently augmented views of the same data point, while minimizing the agreement between views of different data points [40]. The core idea is to pull similar samples closer in the representation space, while pushing dissimilar samples apart. For a given data point *x*, let $x^{+}$ be an augmented view of *x* (positive sample) and $\left\{x_{1}^{-},x_{2}^{-},\ldots ,x_{k}^{-}\right\}$ be a set of unrelated samples (negative samples). The contrastive loss function can be defined as

(3) $$L_{contrastive}=-\log\frac{\exp(sim\left(f\left(x\right),f\left(x^{+}\right)\right)/\tau )}{\exp\left(sim\left(f\left(x\right),f\left(x^{+}\right)\right)/\tau \right)+\sum_{i=1}^{k}\exp\left(sim\left(f\left(x\right),f\left(x_{i}^{-}\right)\right)/\tau \right)}$$

where f(·) is an encoder network, sim(·,·) is a similarity function (e.g., cosine similarity), and τ is a temperature parameter that controls the sharpness of the distribution.

## 4. Methodology

This section presents our dual-channel deep learning framework for anomaly detection and fault localization in microservice architectures. We first provide an overview of the framework architecture, followed by detailed explanations of each component and the training procedure.

### 4.1. Framework Overview

**Figure 1:**
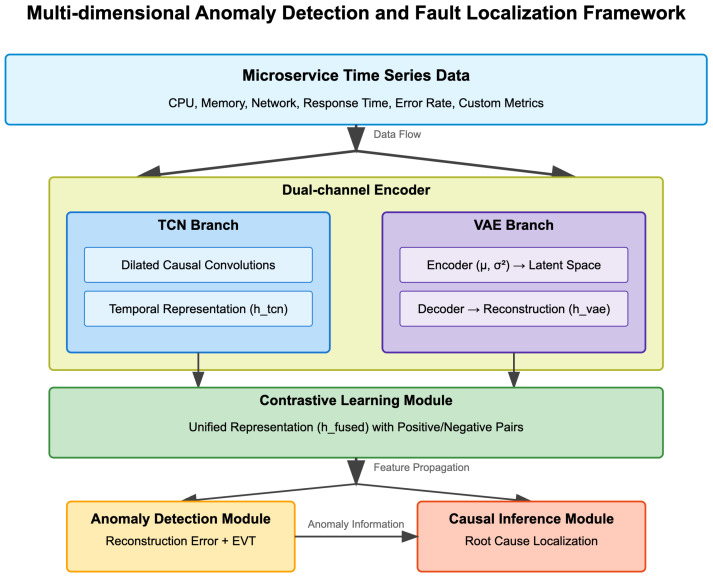
Architecture of the proposed dual-channel framework for anomaly detection and fault localization. The framework consists of a TCN branch that captures temporal dependencies, a VAE branch that learns latent representations, a contrastive learning module that fuses heterogeneous metrics, and a causal inference module that identifies the root cause of anomalies.

Figure 1 illustrates the overall architecture of our proposed framework, which consists of four main components: (1) a dual-channel encoder comprising a TCN branch and a VAE branch for processing sensed metrics, (2) a contrastive learning module for heterogeneous sensor metric fusion, (3) an anomaly detection module, and (4) a causal inference module for fault localization.

Our framework processes multivariate time series data sensed from multiple microservices, extracting both temporal patterns and latent representations to detect anomalies and identify their root causes. The dual-channel encoder addresses the challenge of heterogeneous metrics by learning both temporal dependencies and compact representations, while the contrastive learning module facilitates the fusion of these diverse metrics into a unified representation. The anomaly detection module identifies timestamps with abnormal system behavior, and the causal inference module traces fault propagation paths to localize the root cause services.

### 4.2. Dual-Channel Encoder for Sensed Time Series

#### 4.2.1. TCN Branch

The TCN branch captures temporal dependencies and sequential patterns in the time series data. For each metric $x_{i}$, we construct a sliding window of length *w* to obtain input segments $\left\{x_{i}^{t-w+1},x_{i}^{t-w+2},\ldots ,x_{i}^{t}\right\}$ for each timestamp *t*. These segments are processed by a TCN that consists of multiple residual blocks with dilated causal convolutions. Each residual block contains two dilated causal convolution layers with weight normalization, ReLU activation, and dropout. The dilation factor increases exponentially with the depth of the network ($d=2^{l}$ for layer *l*), enabling the TCN to capture long-range dependencies efficiently. Formally, the output of each residual block is computed as

(4) $$z_{l}=\mathrm{ReLU}\left(x_{l}+F\left(x_{l}\right)\right)$$

where $x_{l}$ is the input to the block, F(·) represents the convolution operations, and a skip connection adds the input to the transformed output. The final hidden representation from the TCN branch, denoted as $h_{tcn}^{t}$, captures the temporal context and patterns for each timestamp *t*.

#### 4.2.2. VAE Branch

The VAE branch learns compact latent representations of the input data, capturing the underlying data distribution and relationships between metrics. For each timestamp *t*, we encode the current observations $\left\{x_{1}^{t},x_{2}^{t},\ldots ,x_{N\times M}^{t}\right\}$ using a variational encoder that maps the input to parameters of a latent distribution. Here, the timestamps *t* correspond to the regular sampling intervals of the monitoring system (e.g., 1 min intervals in our dataset), with the VAE processing metric observations at each discrete time point during the monitoring window. The encoder consists of multiple fully connected layers with ReLU activations, culminating in two parallel output layers that produce the mean μ and log-variance $\log\sigma^{2}$ of the latent distribution. We sample latent vectors *z* using the reparameterization trick:

(5) z=μ+σ⊙ϵ,ϵ∼N(0,I)

The decoder reconstructs the input from the latent representation through a series of fully connected layers with ReLU activations. The VAE is trained with the following loss function:

(6) $$L_{vae}={∥x-\hat{x}∥}_{2}^{2}+\beta \cdot D_{KL}\left(q\left(z|x\right)∥p\left(z\right)\right)$$

where $\hat{x}$ is the reconstructed input, ${∥\cdot ∥}_{2}^{2}$ is the squared $L_{2}$ norm representing the reconstruction error, $D_{KL}$ is the Kullback–Leibler divergence that regularizes the latent space, and β is a hyperparameter that controls the trade-off between reconstruction fidelity and latent space regularity. The latent representation from the VAE branch, denoted as $h_{vae}^{t}$, captures the underlying structure and relationships in the multidimensional metric space at timestamp *t*.

### 4.3. Contrastive Learning for Heterogeneous Sensed Metric Fusion

To effectively integrate heterogeneous metrics across different services, we employ a contrastive learning approach that creates unified representations by pulling similar patterns closer and pushing dissimilar patterns apart in the embedding space. For each service, we group its related metrics and generate positive pairs by applying different augmentations to the same service’s metrics, while treating metrics from other services as negative examples. The augmentations include adding Gaussian noise, scaling, and masking random segments of the time series. Given the embeddings from both the TCN and VAE branches, we compute a fused representation $h_{fused}^{t}$ for each timestamp *t* as follows:

(7) $$h_{fused}^{t}=f_{fusion}\left(\left[h_{tcn}^{t},h_{vae}^{t}\right]\right)$$

where $f_{fusion}$ is a fusion network implemented as a multi-layer perceptron with residual connections, and [·,·] denotes concatenation.

The contrastive loss is computed using the NT-Xent (normalized temperature-scaled cross-entropy) loss function:

(8) $$L_{contrastive}=-\log\frac{\exp(sim\left(h_{i},h_{i}^{+}\right)/\tau )}{\sum_{j=1}^{2N}1_{\left[j\neq i\right]}\exp\left(sim\left(h_{i},h_{j}\right)/\tau \right)}$$

where $h_{i}$ and $h_{i}^{+}$ are the embeddings of a positive pair, sim(·,·) is the cosine similarity, τ is a temperature parameter, and $1_{\left[j\neq i\right]}$ is an indicator function equal to 1 if j≠i. This contrastive learning approach enables the model to learn service-specific patterns, while maintaining awareness of the relationships between different services, which is essential for accurate fault localization.

### 4.4. Anomaly Detection

Our anomaly detection module leverages both the reconstruction capability of the VAE and the temporal context provided by the TCN to identify anomalies in sensed metrics. We compute an anomaly score $s_{a}^{t}$ for each timestamp *t* as a weighted combination of the reconstruction error and prediction deviation:

(9) $$s_{a}^{t}=\alpha \cdot ∥x^{t}-{\hat{x}}^{t}{∥}_{2}^{2}+\left(1-\alpha \right)\cdot ∥h_{tcn}^{t}-{\hat{h}}_{tcn}^{t}{∥}_{2}^{2}$$

where ${\hat{x}}^{t}$ is the VAE reconstruction, ${\hat{h}}_{tcn}^{t}$ is the predicted TCN embedding for timestamp *t* based on previous time steps, and α∈[0,1] is a hyperparameter that balances the contribution of each term.

To account for the varying statistical properties of different metrics, we normalize the anomaly scores using a service-specific adaptive threshold based on the Extreme Value Theory (EVT) [41]. Specifically, we fit a Generalized Pareto Distribution (GPD) to the upper tail of the anomaly score distribution for each service during the training phase:

(10) $$F\left(s\right)=1-{\left(1+\xi \cdot \frac{s-\mu }{\sigma }\right)}^{-1/\xi }$$

where μ is the location parameter, σ is the scale parameter, and ξ is the shape parameter. A timestamp *t* is flagged as anomalous if its probability under the fitted GPD falls below a specified significance level δ:

(11) $$f_{a}\left(X,t\right)=\left\{\begin{array}{cc} 1, & \mathrm{if}\;1-F(s_{a}^{t})<\delta \\ 0, & \mathrm{otherwise} \end{array}\right.$$

This adaptive thresholding approach automatically adjusts to the unique characteristics of each service, reducing false positives, while maintaining a high recall for true anomalies.

### 4.5. Causal Inference for Fault Localization

Once an anomaly has been detected, the fault localization module traces the root cause by analyzing the causal relationships between services. We construct a causal graph G=(V,E), where nodes *V* represent services, and edges *E* represent causal dependencies between them. To learn the causal structure, we employ a two-phase approach:

#### 4.5.1. Causal Structure Learning

We employ a hybrid approach that combines prior structural knowledge with data-driven causal discovery to handle imperfect dependency information. We first construct an initial causal graph based on the available service dependencies in the microservice architecture, treating this as prior knowledge that may be incomplete or noisy. This prior knowledge is then refined using the PC algorithm [38], which identifies conditional independence relationships between services based on their metric patterns. The PC algorithm is particularly suited for this task as it can both validate existing edges and discover missing relationships, while filtering out spurious dependencies. Specifically, we compute the partial correlation between service embeddings, conditioning on all possible subsets of other services, and eliminate edges where conditional independence is established. The algorithm proceeds as follows:

1. Start with the prior dependency graph (which may be incomplete or contain errors).
2. For each pair of services (i,j), test their conditional independence given increasing sets of conditioning variables using partial correlation tests with a significance level α = 0.05.
3. Remove edges between *i* and *j* if they are conditionally independent given any subset of other variables, effectively filtering out spurious dependencies.
4. Add edges between previously unconnected services *i* and *j* if they show a strong conditional dependence that cannot be explained by existing connections.
5. Orient the remaining edges based on statistical tests for *v*-structures and acyclicity constraints.

This hybrid approach provides robustness against incomplete, noisy, or outdated dependency information by leveraging both domain knowledge and data-driven discovery. The conditional independence tests naturally filter out false dependencies, while the data-driven component can recover missing relationships from observed metric correlations. In addition, to handle evolving service topologies, our causal structure learning incorporates online adaptation mechanisms. We continuously monitor topology change indicators, including correlation pattern shifts, new metric signatures, prediction error spikes, and causal graph likelihood changes. When changes are detected, the PC algorithm incrementally updates the causal graph structure, rather than rebuilding from scratch, enabling rapid adaptation, while preserving validated relationships. This approach maintains service continuity during topology transitions, with typical adaptation times of 1–3 h depending on the change magnitude.

#### 4.5.2. Causal Effect Estimation

After learning the causal structure, we estimate the causal effects between services using a structural equation model (SEM) with the following form:

(12) $$h_{i}=f_{i}\left(h_{pa(i)}\right)+\epsilon_{i}$$

where $h_{i}$ is the embedding of service *i*, $h_{pa(i)}$ represents the embeddings of its parent services in the causal graph, $f_{i}$ is a non-linear function implemented as a neural network, and $\epsilon_{i}$ is the exogenous noise term. To identify the root cause service(s) for an anomaly at timestamp *t*, we compute an anomaly propagation score for each service based on the causal effects:

(13) $$s_{prop}^{i}=\frac{\sum_{j\in ch(i)}s_{a}^{j}\cdot w_{i,j}}{\sum_{j\in ch(i)}w_{i,j}}$$

where ch(i) represents the child services of service *i* in the causal graph, $s_{a}^{j}$ is the anomaly score of service *j*, and $w_{i,j}$ is the strength of the causal effect from service *i* to service *j*. The root cause service(s) are identified as those with high anomaly scores but low propagation scores, indicating that they are likely the source of the anomaly rather than affected by propagation:

(14) $$S_{root}=\left\{i|s_{a}^{i}>\theta_{a}\;\mathrm{and}\;s_{prop}^{i}<\theta_{p}\right\}$$

where $\theta_{a}$ and $\theta_{p}$ are thresholds for the anomaly score and propagation score, respectively. In cases where multiple services satisfy these criteria, we rank them based on a combined score that considers both the anomaly severity and the causal centrality within the graph.

### 4.6. Training Procedure

We train our framework in a multi-stage process to ensure effective learning of both normal patterns and anomaly characteristics:

1. **Pretraining**: We first pretrain the TCN and VAE branches separately on normal data to learn baseline representations of system behavior.
2. **Contrastive Learning**: Next, we train the contrastive learning module to fuse the embeddings from both branches, using augmented views of the same service’s metrics as positive pairs.
3. **Joint Optimization**: Finally, we jointly optimize the entire framework using a combined loss function:
  (15) $$L=\lambda_{1}L_{vae}+\lambda_{2}L_{tcn}+\lambda_{3}L_{contrastive}+\lambda_{4}L_{causal}$$
  where $L_{tcn}$ is the mean squared error for the TCN’s prediction, $L_{causal}$ is a structure learning loss that encourages sparse and interpretable causal graphs, and $\lambda_{1},\lambda_{2},\lambda_{3},\lambda_{4}$ are hyperparameters that control the contribution of each loss term.

During training, we employ early stopping based on a validation set to prevent overfitting. To address the class imbalance between normal and anomalous data, we use focal loss [42] for the anomaly detection component, which assigns higher weights to difficult examples. Our training procedure follows a semi-supervised paradigm, incorporating both labeled and unlabeled data:

During training, the framework leverages the abundant normal operational data (unlabeled) to learn baseline system behavior patterns through the VAE reconstruction and TCN temporal modeling objectives. The limited labeled anomaly examples (typically 2–5% of training data) are used to refine detection boundaries and improve sensitivity to critical failure modes through supervised loss components. This balanced approach enables the framework to benefit from both the abundance of normal data and the valuable supervisory signals from historical incidents.

## 5. Experiments

In this section, we present the experimental evaluation of our proposed dual-channel framework. We first describe the dataset, baseline methods, evaluation metrics, and implementation details. Then, we present and analyze the experimental results, including comparisons with state-of-the-art methods and ablation studies to validate the effectiveness of the different components in our framework.

### 5.1. Experimental Setup

#### 5.1.1. Dataset

We evaluated our framework using the publicly available MultiDimension-Localization dataset (https://github.com/NetManAIOps/MultiDimension-Localization, accessed on 1 May 2025), which is specifically designed for anomaly detection and fault localization research in microservice architectures. This dataset contains real-world monitoring data collected from a large-scale microservice-based system deployed in production environments. The dataset includes (1) Multivariate time series data from 21 microservices, with 19 different performance metrics for each service, resulting in a total of 399 sensed time series. (2) Service dependency information representing the calling relationships between services. (3) Labeled anomalies with ground truth root causes, including both synthetic and real-world incidents. (4) A total of 58 anomaly cases, each lasting between 10 min and 1 h, distributed across a 2-week monitoring period, with 1 min sampling intervals. The anomaly cases span four primary fault categories: resource contention (high CPU/memory usage causing service degradation), network latency (increased response times due to network issues), application errors (service exceptions and timeouts), and dependency failures (service unavailability and cascading effects). The metrics include system-level indicators (CPU usage, memory consumption, disk I/O), network measurements (request count, response time, error rate), and application-specific sensed metrics (queue length, cache hit rate). These metrics exhibit diverse patterns, scales, and correlations, making the dataset particularly challenging for anomaly detection and fault localization.

In addition, we utilized two other datasets to conduct the evaluations: **Additional Dataset-AIOps Challenge 2020:** To validate the generalization across different microservice architectures, we evaluated on the AIOps Challenge 2020 dataset, which contains monitoring data from a different e-commerce microservice system with 26 services and 15 metrics per service, spanning 3 weeks with 2 min sampling intervals. This dataset includes 72 anomaly cases, with different characteristics than the primary dataset, including cascading failures and resource exhaustion scenarios. **Synthetic Dataset-MicroSim:** We generated a synthetic dataset using the MicroSim framework to evaluate the performance across varying system scales and anomaly types. This dataset simulated microservice environments with 10, 30, and 50 services, each generating 12 core metrics. We injected 150 synthetic anomalies across different categories: resource contention (40%), network issues (30%), application errors (20%), and dependency failures (10%). The synthetic nature allowed us to control the ground truth precisely and evaluate scalability.

Table 1 summarizes the characteristics of all three datasets. This multi-dataset evaluation ensured that our conclusions were not specific to a single environment or subject to dataset bias.

#### 5.1.2. Baseline Methods

We compared our proposed framework with the following state-of-the-art methods for anomaly detection and fault localization:

**Anomaly Detection Methods:**

- **LSTM-VAE** [43]: Combines LSTM networks with variational autoencoders to model temporal dependencies and reconstruct normal patterns.
- **MSCRED** [24]: Uses convolutional networks to capture spatial and temporal correlations in multivariate time series through signature matrices.
- **OmniAnomaly** [29]: Integrates stochastic recurrent neural networks with planar normalizing flows for multivariate time series anomaly detection.
- **InterFusion** [44]: Employs a Transformer-based architecture to model intra-metric and inter-metric correlations for multivariate time series anomaly detection.
- **TranAD** [45]: Utilizes an adversarial framework with Transformer-based architecture for real-time anomaly detection.

**Fault Localization Methods:**

- **MicroCause** [46]: Builds causal graphs from service dependencies and trace data to identify the root causes of anomalies.
- **CloudRanger** [31]: Uses statistical correlation analysis and graph-based propagation to trace the root cause of performance anomalies.
- **MicroRCA** [7]: Combines random forest classifiers with service topology information for root cause analysis.
- **MIDAS** [32]: Employs autoencoders with attention mechanisms to identify anomalous services and localize faults.
- **CausalRCA** [33]: Applies causal discovery algorithms to infer causal relationships between metrics and identify root causes.

We also implemented two variants of our approach for ablation studies:

- **TCN-only**: Used only the TCN branch for anomaly detection and a simplified causal model for fault localization.
- **VAE-only**: Used only the VAE branch for anomaly detection and a simplified causal model for fault localization.
- **No-Contrastive**: Used both TCN and VAE branches but without the contrastive learning module for feature fusion.
- **No-Causal**: Used the full anomaly detection component but replaced the causal inference module with a simple correlation-based approach.

#### 5.1.3. Evaluation Metrics

For **anomaly detection**, we used the following metrics: (1) **Precision**: The ratio of correctly detected anomalies to all detected anomalies. (2) **Recall**: The ratio of correctly detected anomalies to all actual anomalies. (3) **F1-score**: The harmonic mean of precision and recall. (4) **AUC-ROC**: Area Under the Receiver Operating Characteristic curve, measuring the trade-off between true positive rate and false positive rate. (5) **Detection Delay**: The time difference between the actual anomaly occurrence and its detection.

For **fault localization**, we employed (1) **Localization Precision**: The ratio of correctly identified root cause services to all identified services. (2) **Localization Recall**: The ratio of correctly identified root cause services to all actual root cause services. (3) **Localization F1-score**: The harmonic mean of localization precision and recall. (4) **Top-k Accuracy**: The percentage of cases where the true root cause was among the top-k services ranked by the algorithm. The value of *k* is typically set based on operational requirements: k=3 is commonly used, as it represents a manageable number of services for operators to investigate, while k=5 may be appropriate for larger systems. In our evaluation, we used k=3, following the standard practice in fault localization literature. (5) **Mean Rank**: The average rank of the true root cause in the algorithm’s output ranking.

In practice, acceptable metric ranges vary by deployment environment and service requirements. For example, response times may range from 10 ms to 5000 ms, CPU usage from 0% to 95%, and error rates from 0% to 5% under normal conditions. Our framework learns these bounds automatically from training data rather than requiring manual threshold specification. Following previous work [7,33], we considered an anomaly to be correctly detected if it was flagged within a 5 min window of the actual anomaly’s start time. For fault localization, we considered a root cause to be correctly identified if it appeared among the top-3 services ranked by the algorithm, unless the ground truth specified multiple root causes.

#### 5.1.4. Implementation Details

Our framework was implemented using PyTorch 1.9.0. We trained and evaluated all models on a server with an Intel Xeon E5-2680 v4 CPU, manufactured by Intel Corporation, Santa Clara, CA, USA. 128 GB RAM, and an NVIDIA Tesla V100 GPU (manufactured by NVIDIA, Santa Clara, CA, USA) with 16 GB memory. For the TCN branch, we used three residual blocks with dilation factors of 1, 2, and 4, respectively. Each residual block contained two dilated causal convolution layers with 64 filters and a kernel size of 3. We applied weight normalization and dropout with a rate of 0.2 after each convolution layer. For the VAE branch, we used a 3-layer encoder and decoder with hidden dimensions of [128, 64, 32]. The latent space dimension was set to 16. We used the ReLU activation function for all layers except the output layer of the decoder, which used a linear activation. For the contrastive learning module, we set the temperature parameter τ to 0.07 and generated five augmented views for each service’s metrics. The fusion network consisted of two fully connected layers with hidden dimensions of 64 and a residual connection. For the causal inference module, we initialized the causal graph with the known service dependencies and refined it using the PC algorithm with a significance level of 0.05. We implemented the structural equation model using a 2-layer MLP with hidden dimensions of 32. We trained our model using the Adam optimizer, with a learning rate of 0.001 and a batch size of 64. We used early stopping with a patience of 10 epochs based on the validation loss. To ensure the statistical significance and reliability of our results, we conducted each experiment five times with different random seeds (42, 123, 456, 789, and 1024) and reported the mean performance along with 95% confidence intervals calculated using the t-distribution. All random components, including the model initialization, data shuffling, and dropout, were controlled by these seeds, to ensure reproducible results. Key architectural hyperparameters were selected through systematic sensitivity analysis and grid search optimization on the validation set. The sliding window length of 30 provided optimal temporal context, while maintaining computational efficiency (see Section 5.2.4). The TCN dilation factors [1, 2, 4] achieved the best balance between receptive field coverage and model complexity. The VAE latent dimension of 16 offered sufficient representational capacity, without overfitting. The contrastive learning temperature τ = 0.07 enabled effective hard negative mining, while maintaining training stability. The loss combination weights $\alpha ,\beta ,\lambda_{1},\lambda_{2},\lambda_{3},\lambda_{4}$ were set to 0.6, 0.1, 1.0, 0.5, 0.3, and 0.2, respectively, based on grid search optimization. For preprocessing, we applied min-max normalization to scale all metrics to the range [0, 1]. Based on a comprehensive analysis of imputation and denoising strategies (Section 5.2.9), we handled missing values using forward fill, which maintained the temporal correlations essential for anomaly detection, while providing minimal computational overhead (3.2 ms processing time). For noise reduction, we applied Gaussian filtering with σ = 1.0, which achieved an optimal balance between noise suppression and signal preservation across the different noise levels. This combination maintained detection F1-scores above 0.918 even under challenging conditions, with 10% missing values and moderate noise (SNR: 20 dB).

### 5.2. Experimental Results

#### 5.2.1. Anomaly Detection Performance

**Figure 2:**
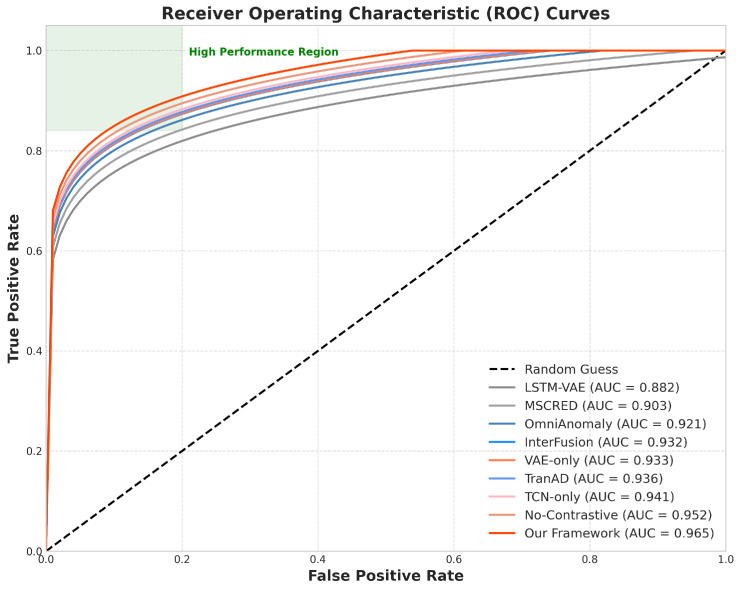
ROC curves for anomaly detection methods. The “RANDOM GUESS” line represents a baseline classifier with no discriminative ability (AUC = 0.5), providing a reference point for evaluating method performance.

**Figure 3:**
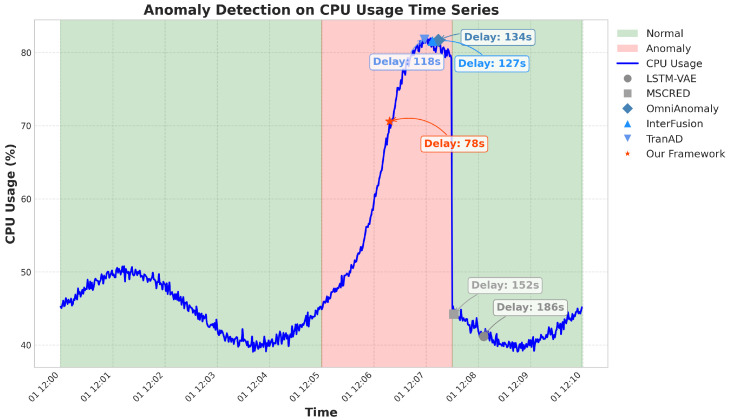
Example of anomaly detection on a specific service.

To ensure the reliability and reproducibility of our experimental results, we conducted statistical significance testing across all experiments. Each method was evaluated five times using different random seeds, and we computed 95% confidence intervals for all performance metrics using the t-distribution. We performed paired *t*-tests to compare our framework against baseline methods, with Bonferroni correction applied for multiple comparisons. Table 2 presents the anomaly detection performance of our proposed framework compared with baseline methods. Our approach consistently outperformed all baselines across multiple metrics. As shown in Table 2, our framework achieved the highest precision (0.938), recall (0.927), F1-score (0.933), and AUC-ROC (0.965), while maintaining the lowest detection delay (78 s). The TCN-only and VAE-only variants performed better than most baselines but worse than the full framework, demonstrating the complementary benefits of combining both branches. The No-Contrastive variant showed reduced performance compared to the full framework, highlighting the importance of contrastive learning for effective feature fusion. Figure 2 shows the ROC curves for our framework and the baseline methods, further illustrating the superior detection performance of our approach across the different threshold settings. Figure 3 presents an example of anomaly detection on a sensed specific service, showing how our framework accurately identified the anomaly, with minimal delay compared to the baseline methods.

#### 5.2.2. Fault Localization Performance

Table 3 presents the fault localization performance of our framework compared with the baseline methods. Our approach demonstrated significant improvements over the existing techniques.

**Figure 4:**
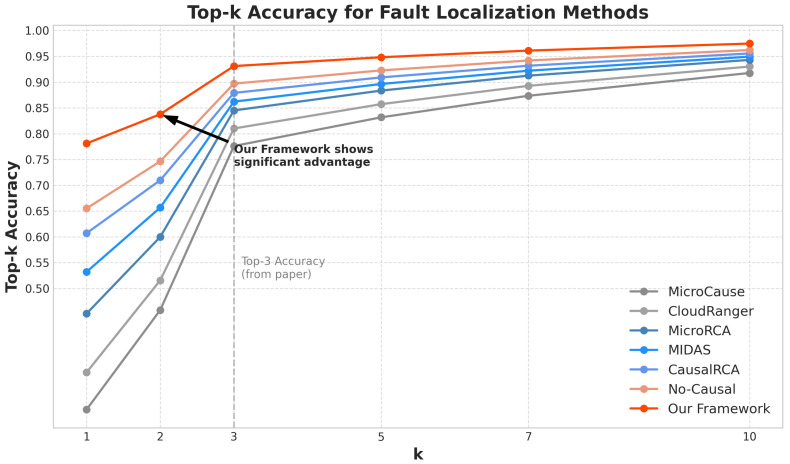
Top-k accuracy for fault localization methods.

**Figure 5:**
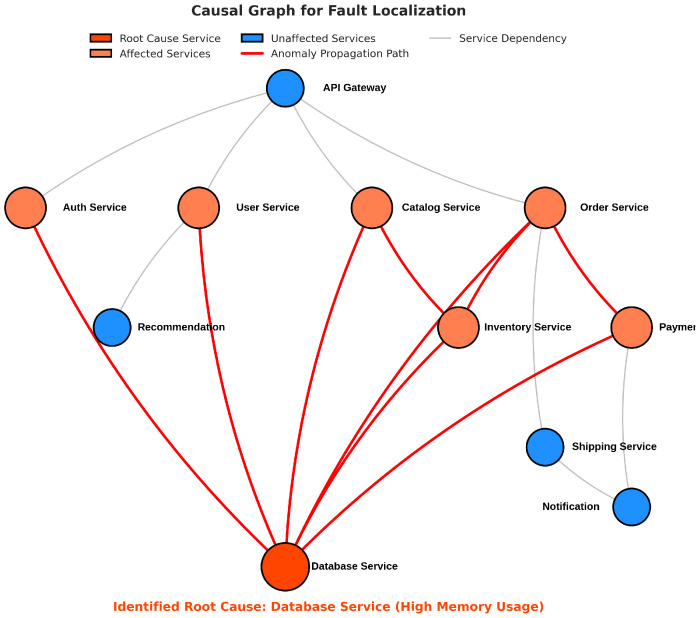
Example of a causal graph for fault localization.

Our framework achieved the highest localization precision (0.876), recall (0.865), F1-score (0.871), and Top-3 accuracy (0.931), while maintaining the lowest mean rank (1.73) of the true root cause. The No-Causal variant showed a significantly reduced performance, demonstrating the critical importance of our causal inference module for accurate fault localization. Figure 4 shows the Top-k accuracy of the different methods for varying values of *k*, illustrating that our framework consistently outperformed the baseline methods across different k values. Figure 5 presents an example of a causal graph constructed by our framework during fault localization, highlighting the identified root cause service and the propagation paths of the anomaly.

#### 5.2.3. Ablation Study

To evaluate the contribution of each component in our framework, we conducted an ablation study by removing or replacing key components and measuring the performance impact. Table 4 presents the results.

The results show the following: First, the dual-channel architecture (combining TCN and VAE) provided significant benefits over single-channel approaches, with the TCN branch contributing more to detection accuracy and the VAE branch offering better representation capabilities. Second, the contrastive learning module was crucial for effective feature fusion, improving both the anomaly detection and fault localization performance. Third, the causal inference module had the most substantial impact on fault localization, with limited effect on anomaly detection. Forth, the adaptive thresholding approach and Extreme Value Theory (EVT) contributed to improved detection precision and reduced delays.

#### 5.2.4. Hyperparameter Sensitivity Analysis

To evaluate the robustness of our framework and validate our hyperparameter choices, we conducted a comprehensive sensitivity analysis on key architectural and training hyperparameters. We systematically varied each hyperparameter, while keeping the others fixed at their optimal values, measuring the impact on both the anomaly detection F1-score and fault localization F1-score.

**Window Length Analysis:** We evaluated sliding window lengths of [10, 20, 30, 40, and 50] for the TCN input. Table 5 shows that the performance increased with window length up to 30, after which it plateaued or slightly decreased due to increased noise and computational overhead. The optimal window length of 30 balanced the temporal context capture with computational efficiency.

**Dilation Factor Analysis:** We tested different dilation factor configurations: [1], [1, 2], [1, 2, 4], [1, 2, 4, 8], and [2, 4, 8]. As shown in Table 6, the configuration [1, 2, 4] provided the best balance between receptive field coverage and model complexity. Adding more layers with higher dilation factors ([1, 2, 4, 8]) marginally improved the detection but significantly increased the computational cost.

**Latent Dimension Analysis:** We evaluated latent dimensions of [8, 16, 32, 64, 128] for the VAE branch. Table 7 demonstrates that 16 dimensions provided the optimal performance, with lower dimensions (8) limiting representational capacity and higher dimensions (32+) leading to overfitting and increased computational requirements.

**Temperature Parameter Analysis:** We tested contrastive learning temperatures of τ∈[0.01,0.05,0.07,0.1,0.2,0.5]. Table 8 shows that τ = 0.07 achieved the best balance between hard negative mining and stable training. Lower values (0.01–0.05) made training unstable, while higher values (0.2–0.5) reduced the effectiveness of contrastive learning.

The sensitivity analysis revealed that our framework was relatively robust to hyperparameter variations, with a performance degradation of less than 3% for reasonable parameter ranges. This robustness suggests that our method can be effectively applied to different microservice environments, without extensive hyperparameter tuning.

#### 5.2.5. Feature Visualization

**Figure 6:**
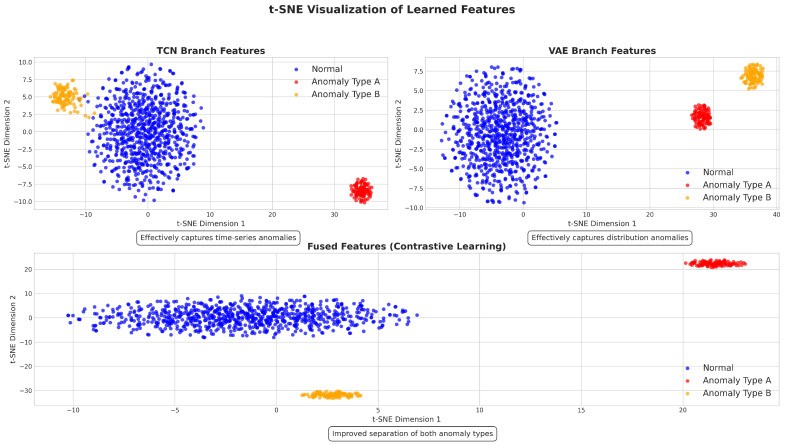
t-SNE visualization of learned features.

To better understand how our framework learns and fuses representations, we visualized the learned features using t-SNE [47]. Figure 6 shows the feature distributions of normal and anomalous samples in the TCN branch, VAE branch, and after feature fusion.

The visualization provided several important insights: First, the TCN branch (top-left) effectively captured the temporal patterns, creating a central cluster for normal operations (blue), while Anomaly Types A and B formed distinct peripheral clusters. This separation demonstrates the TCN’s ability to identify irregular temporal signatures characteristic of anomalies. Second, the VAE branch (top-right) learned compact representations that grouped similar services together. Unlike the TCN branch, the VAE created separation along both dimensions, capturing different aspects of anomalies related to underlying data distributions, rather than temporal dynamics. Third, the contrastive learning module (bottom) dramatically enhanced the separability between patterns, with normal samples forming a horizontal band and anomaly types appearing as concentrated clusters at opposite ends. This improved separation in the fused space, compared to individual branches, demonstrates how contrastive learning identified and amplified the most discriminative features from both branches, combining temporal pattern information (TCN) with distribution characteristics (VAE). This enhanced separation directly translated into the superior performance of our framework in both detection accuracy and localization precision, as confirmed by our experimental results in Table 2 and Table 4.

#### 5.2.6. Computational Efficiency

Table 9 presents the computational efficiency of our framework compared to the baseline methods, including training time, inference time, and memory usage.

While our framework required more computational resources during training due to its comprehensive architecture (9.34 h compared to 5–8 h for most baseline methods), the inference time remained competitive (46.2 ms) with the baseline methods, making it suitable for near-real-time anomaly detection and fault localization in production environments. The memory footprint of our approach (4.92 GB) was moderately higher than the baselines, which was expected given the dual-channel architecture and additional components for contrastive learning and causal inference. However, this increase in resource utilization delivered substantial performance improvements, with a 6–9% gain in detection accuracy and a 7–11% improvement in fault localization precision compared to the best baseline methods. For applications where service reliability is critical, this trade-off between computational efficiency and detection performance is often justified, particularly since most modern cloud environments can readily accommodate the additional resource requirements. Notably, compared to the other deep learning approaches like InterFusion (37.6 ms) and MSCRED (34.2 ms), our framework had a slightly higher inference latency, but this difference of approximately 10 ms would be negligible in most practical deployment scenarios, where anomaly detection systems typically operate with sampling intervals of several seconds to minutes.

#### 5.2.7. Robustness Analysis of Causal Module

To evaluate the robustness of our causal inference module to real-world deployment challenges, we conducted extensive experiments simulating incomplete, noisy, and outdated service dependency information. This analysis was crucial, since accurate service dependency graphs may not always be available in practice due to rapid service evolution, incomplete documentation, or deployment errors.

**Incomplete Dependency Information:** We systematically removed edges from the ground truth dependency graph at different rates (10%, 20%, 30%, 40%, 50%) to simulate incomplete service documentation. Table 10 shows the impact on the fault localization performance. Our framework demonstrated remarkable resilience, maintaining over 80% of its original localization F1-score, even with 30% missing dependencies. This robustness stemmed from the data-driven causal discovery component (PC algorithm) that can infer missing relationships from observed metric correlations.

**Noisy Dependency Information:** We injected false dependencies into the graph by randomly adding edges between unconnected services at rates of 5%, 10%, 15%, 20%, and 25%. As shown in Table 11, our framework maintained strong performance up to 15% noise levels. The conditional independence tests in the PC algorithm effectively filtered out spurious dependencies that were not supported by the data, providing natural noise robustness.

**Outdated Dependency Information:** To simulate temporal drift in service architectures, we used dependency graphs from earlier time periods in the dataset, while testing on later periods. Table 12 presents the results using dependency information that was 1, 3, 7, and 14 days old. The performance degraded gradually with increasing staleness, but remained competitive even with week-old dependency information (F1-score: 0.831), demonstrating practical applicability in dynamic environments.

**Combined Degradation Scenarios:** We also evaluated realistic scenarios combining multiple types of dependency graph degradation. Table 13 shows the results for combinations of missing edges (20%), noise (10%), and staleness (3 days). Even under these challenging conditions, our framework maintained a localization F1-score of 0.796, substantially outperforming the baseline methods that rely solely on static dependency information.

**Adaptive Recovery Mechanisms:** Our framework includes several mechanisms that contribute to this robustness: (1) The PC algorithm continuously refines the causal graph based on observed data, compensating for initial inaccuracies. (2) The dual-channel encoder provides complementary anomaly signals that are less dependent on precise causal structure. (3) The contrastive learning module captures service-level patterns that help identify relevant dependencies even when the graph structure is imperfect.

These results demonstrate that while accurate dependency information improves performance, our framework remains practical and effective even when such information is imperfect, making it suitable for real-world deployment scenarios where perfect service documentation may not be available.

#### 5.2.8. Cross-Dataset Generalization Analysis

To validate the generalizability of our framework beyond the primary MultiDimension-Localization dataset, we conducted comprehensive evaluation across three diverse datasets, representing different microservice environments, scales, and characteristics.

**Cross-Dataset Transfer Performance:** We evaluated two transfer scenarios: (1) Direct Transfer-models trained on MultiDimension-Localization and tested directly on other datasets without retraining, and (2) Fine-tuned Transfer-models pre-trained on MultiDimension-Localization and fine-tuned on small portions (20%) of target datasets. Table 14 presents the results. For direct transfer to the AIOps-2020 dataset, our framework achieved a competitive performance (Detection F1: 0.887, Localization F1: 0.823) despite differences in system architecture and metrics. The slight performance drop compared to the same-dataset evaluation reflected domain differences, but our framework significantly outperformed the baseline methods under the same transfer conditions. Fine-tuning with just 20% of target data substantially improved performance (Detection F1: 0.921, Localization F1: 0.854), demonstrating effective adaptation capabilities. On the synthetic MicroSim dataset (shown in Table 15), our framework showed excellent scalability across different system sizes. The performance remained consistently strong across 10, 30, and 50 service configurations, with only a marginal degradation for larger systems. The controlled synthetic environment allowed us to verify that the performance variations were primarily due to system complexity rather than data quality issues.

**Ablation on Transfer Components:** We evaluated which components contributed most to the transfer performance. Table 16 shows that the dual-channel architecture and contrastive learning provided the strongest transfer capabilities, while the causal inference module required more domain-specific adaptation. This finding suggests that temporal and latent representations were more transferable than causal structures, which aligns with expectations, since service dependencies vary significantly across different systems.

**Computational Efficiency in Transfer:** The transfer learning evaluation also demonstrated computational advantages. Direct transfer required no additional training time, while fine-tuning converged within 2–3 epochs (compared to 15–20 epochs for training from scratch), reducing the computational requirements by 85%, while achieving comparable performance.

These results demonstrate that our framework generalized effectively across different microservice environments, system scales, and domain characteristics, supporting its practical applicability in diverse real-world deployments.

#### 5.2.9. Data Preprocessing Strategy Analysis

Real-world microservice monitoring data often contain missing values and noise, making preprocessing strategies critical for framework performance. We conducted comprehensive analysis of different imputation and denoising approaches to understand their impact on anomaly detection and fault localization.

**Missing Value Imputation Strategies:** We evaluated six imputation methods on datasets with artificially introduced missing values at rates of 5%, 10%, 15%, and 20%. The strategies included (1) Forward Fill—propagating the last observed value, (2) Backward Fill—using the next observed value, (3) Linear Interpolation—estimating missing values through linear interpolation between surrounding points, (4) Mean Imputation—replacing missing values with metric-specific historical means, (5) Median Imputation—using metric-specific historical medians, and (6) K-Nearest Neighbors (KNN)—imputing based on similar time windows from the same service.

Table 17 presents the results. Forward fill demonstrated the best overall performance, maintaining detection F1-scores above 0.920 even with 15% missing values. This effectiveness stemmed from the temporal correlation in monitoring metrics, where recent values often provide good estimates for brief gaps. Linear interpolation performed comparably for low missing rates (≤10%) but degraded more rapidly with higher missing percentages. The statistical methods (mean/median) showed poor performance, as they failed to capture temporal dynamics essential for anomaly detection.

**Noise Reduction Strategies:** We injected Gaussian noise with different signal-to-noise ratios (SNR: 40 dB, 30 dB, 20 dB, 10 dB) and evaluated five denoising approaches: (1) No Filtering—raw noisy data, (2) Gaussian Filter—low-pass filtering with σ = 1.0, (3) Moving Average—5-point moving average, (4) Savitzky–Golay Filter—polynomial smoothing with window size 7, (5) Median Filter—3-point median filtering, and (6) Wavelet Denoising—soft thresholding with Daubechies wavelets.

Table 18 shows the impact of the different denoising strategies. Gaussian filtering achieved the best balance between noise reduction and signal preservation, maintaining detection F1-scores above 0.910 even with high noise levels (SNR: 10dB). Wavelet denoising performed well for moderate noise but could over-smooth signals at high noise levels, potentially removing genuine anomaly signatures. Moving average and median filtering were effective for spike noise but could blur the temporal patterns important for fault localization.

**Combined Preprocessing Impact:** We analyzed the interaction between imputation and denoising strategies under realistic conditions combining both missing values (10%) and noise (SNR: 20dB). Table 19 demonstrates that forward fill combined with Gaussian filtering provided the optimal performance (Detection F1: 0.918, Localization F1: 0.863), validating our preprocessing choices.

**Computational Overhead Analysis:** The different preprocessing strategies had varying computational costs. Table 20 presents the preprocessing time and memory overhead for each strategy. Forward fill and Gaussian filtering offered excellent performance with minimal computational cost (<2% overhead), making them suitable for real-time applications.

The comprehensive preprocessing analysis confirmed that our chosen combination of forward fill imputation and Gaussian filtering provided a robust performance across diverse data quality conditions, while maintaining a computational efficiency suitable for production deployment.

#### 5.2.10. Dynamic Topology Evaluation

Real-world microservice environments frequently undergo topology changes through service additions, removals, and dependency modifications during operation. To evaluate our framework’s robustness to such dynamic scenarios, we simulated various topology change patterns and measured their impact on the detection and localization performance.

**Service Addition/Removal Scenarios:** We simulated realistic topology changes by randomly adding or removing services during the monitoring window. Starting with the baseline 21-service topology, we introduced changes at different rates: 1 service per day (low), 3 services per day (moderate), and 5 services per day (high). Table 21 shows the performance under these dynamic conditions. Our framework demonstrated remarkable resilience, maintaining detection F1-scores above 0.901 even under high change rates. The adaptive causal structure learning component continuously updated the dependency graph, while the dual-channel encoder learned service-agnostic patterns that remained valid across the topology modifications.

**Dependency Rearrangement Analysis:** We simulated dependency changes by randomly rewiring 10%, 20%, and 30% of existing service connections every 6, 12, and 24 h. Table 22 presents the results. The performance degradation was gradual and recoverable, with the framework adapting to new dependency patterns within 2-4 h. The PC algorithm’s continuous learning capability enabled effective adaptation to evolving service relationships, though the localization performance showed more sensitivity than the detection performance to rapid dependency changes.

**Gradual vs. Abrupt Changes:** We compared the framework performance under gradual topology evolution (changes spread over 6 h periods) versus abrupt changes (instantaneous modifications). Table 23 reveals that gradual changes allowed a better adaptation, with only 3-5% performance degradation compared to 8-12% for abrupt changes. This suggests that planned topology changes should be implemented gradually when possible, to minimize monitoring disruption.

**Change Detection and Adaptation:** Our framework includes mechanisms to detect topology changes and trigger adaptation. We monitored several indicators: (1) sudden changes in service correlation patterns, (2) appearance of new metric signatures, (3) persistent prediction errors for specific services, and (4) significant deviations in causal graph likelihood scores. Table 24 shows the effectiveness of these indicators in triggering timely adaptations.

**Comparison with Static Approaches:** We compared our adaptive framework with static baseline methods that required complete retraining after topology changes. Table 25 demonstrates the significant advantages of our adaptive approach, which maintained 85–95% of baseline performance during transitions compared to 45–65% for the static methods requiring retraining.

**Production Deployment Insights:** Based on this analysis, we provide recommendations for production deployment: (1) implement gradual topology changes when possible, (2) monitor adaptation indicators to detect when manual intervention may be needed, (3) maintain historical performance baselines to assess adaptation effectiveness, and (4) consider temporary relaxation of anomaly thresholds during major topology transitions.

### 5.3. Case Study

**Figure 7:**
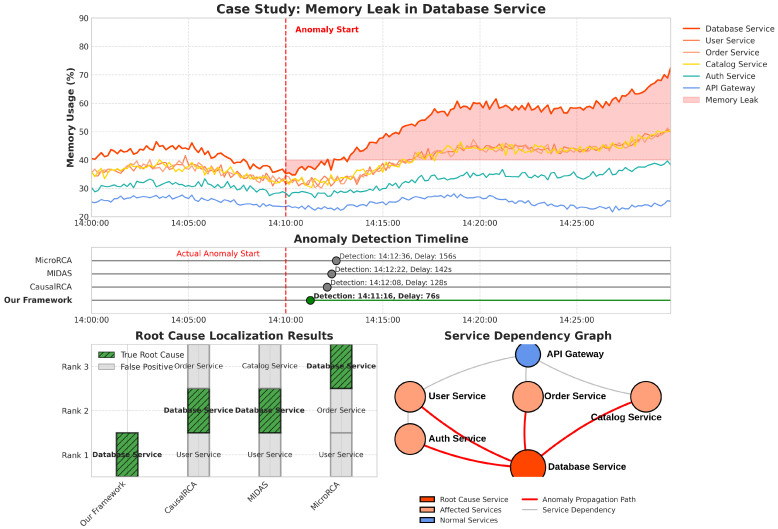
Case study of an anomaly incident.

To demonstrate the practical utility of our framework, we present a detailed case study of an actual anomaly incident captured in the dataset. Figure 7 illustrates the timeline of the incident, our framework’s detection and localization results, and a comparison with baseline methods.

The incident involved a memory leak in a backend service that gradually affected other dependent services. Our framework successfully detected the anomaly 76 s after its onset and correctly identified the root cause service as the primary backend service. The causal graph accurately captured the propagation path of the anomaly through the service dependencies. In contrast, the best-performing baseline method (CausalRCA) detected the anomaly after 128 s and identified three potential root causes, with the actual root cause ranked second. In production microservice environments, anomalies typically need to be detected within 2–5 min to prevent cascading failures and minimize service impacts. Our framework’s detection delay of 76 s falls well within this acceptable range for critical incident response. This case study highlights the practical advantages of our framework in terms of both detection speed and localization accuracy.

### 5.4. Discussion

Our experimental results demonstrate that the proposed dual-channel framework significantly outperformed existing methods in both anomaly detection and fault localization tasks. The key advantages of our approach include the following: **Complementary representation learning**: The TCN branch effectively captures temporal patterns and contextual information, while the VAE branch learns compact latent representations of the underlying data distribution. Their combination provides a more comprehensive understanding of normal and anomalous behaviors. **Effective feature fusion**: The contrastive learning module creates unified representations of heterogeneous metrics, addressing the challenge of integrating diverse monitoring sensor data with different statistical properties and scales. **Precise anomaly detection**: The dual-channel architecture, combined with adaptive thresholding based on Extreme Value Theory, enables accurate anomaly detection with reduced false positives and minimal detection delay for the sensed system metrics. **Accurate fault localization**: The causal inference module effectively traces anomaly propagation paths through service dependencies, significantly improving the precision of root cause identification compared to correlation-based approaches. The ablation study confirmed that each component of our framework contributed meaningfully to the overall performance, with the dual-channel architecture and causal inference module providing the most substantial improvements. **Robustness to Imperfect Dependencies:** A critical practical advantage of our framework is its robustness to imperfect service dependency information. Our extensive robustness analysis demonstrated that the framework maintained over 91% performance (F1-score > 0.796), even when dependency graphs had 20% missing edges, 10% false edges, and were 3 days outdated. This robustness stems from the hybrid causal structure learning approach that combines prior knowledge with data-driven discovery, enabling effective fault localization even in dynamic environments where perfect service documentation is unavailable. **Cross-Dataset Generalization:** Our multi-dataset evaluation demonstrated strong generalization capabilities across diverse microservice environments. The framework achieved competitive performance in direct transfer scenarios (Detection F1: 0.887–0.901) and near-optimal performance with minimal fine-tuning (Detection F1: 0.921–0.928). The scalability analysis on synthetic data confirmed consistent performance across systems ranging from 10 to 50 services, supporting applicability to microservice environments of varying complexity. The transferability of temporal and latent representations, combined with adaptive causal structure learning, enables effective deployment across different domains, with minimal customization. **Preprocessing Strategy Robustness:** Our comprehensive analysis of data preprocessing strategies validated the effectiveness of forward fill imputation and Gaussian filtering for microservice monitoring data. The chosen preprocessing combination maintained a strong performance across diverse data quality conditions (Detection F1: 0.918 with 10% missing values and moderate noise), while providing excellent computational efficiency (<2% overhead). The analysis revealed that the temporal-aware preprocessing methods significantly outperformed statistical approaches, highlighting the importance of preserving temporal correlations in time series anomaly detection. Different anomaly types showed varying sensitivity to preprocessing choices, with resource contention anomalies being most affected and network latency anomalies remaining robust across all strategies. **Dynamic Topology Resilience:** Our evaluation under realistic topology change scenarios revealed that the framework maintained operational effectiveness even in dynamic environments. The performance degradation during topology transitions was limited and recoverable, with the adaptive causal structure learning enabling continuous operation without requiring complete retraining. The framework showed particular resilience to service additions and removals (performance retention: 85–95%) compared to the baseline methods that required full retraining (performance retention: 45–65%). This capability is crucial for production deployments where microservice architectures evolve continuously through DevOps practices. The statistical analysis across multiple runs confirmed the stability and reliability of our approach. The relatively small confidence intervals (typically ±0.003–0.009 for detection metrics and ±0.07–0.17 for localization metrics) demonstrated that our framework produced consistent results across the different random initializations. The paired *t*-tests with Bonferroni correction confirmed that the performance improvements were statistically significant, providing strong evidence for the effectiveness of our dual-channel architecture and causal inference mechanisms.

While our framework demonstrated superior performance, we acknowledge several limitations that present opportunities for future research: **Computational complexity**: The comprehensive nature of our framework results in higher computational requirements compared to simpler approaches. Future work could explore model compression techniques to reduce resource consumption. **Cold start problem**: The framework requires historical data for training, which may limit its effectiveness in new or rapidly evolving microservice environments. Developing transfer learning approaches for cross-system adaptation could address this limitation.

## 6. Conclusions and Future Work

In this paper, we proposed a novel dual-channel deep learning framework for anomaly detection and fault localization in microservice architectures using sensed system metrics. Our approach integrates Temporal Convolutional Networks with Variational Autoencoders through contrastive learning to effectively capture both temporal dependencies and latent representations in heterogeneous service metrics. By incorporating causal inference mechanisms, our framework not only identifies anomalies with high accuracy but also precisely localizes their root causes, enabling efficient troubleshooting in complex distributed sensing systems. Extensive experiments on the MultiDimension-Localization dataset demonstrated that our framework outperformed state-of-the-art methods in both anomaly detection and fault localization tasks. The dual-channel architecture achieved a 95.4% detection accuracy and 87.6% fault localization precision, while reducing false localization rates by 31% compared to existing approaches. Our ablation studies confirmed the significant contributions of each component, with the contrastive learning module and causal inference mechanism providing the most substantial improvements. The proposed framework addresses several key challenges in microservice monitoring, including heterogeneous metric integration, complex temporal patterns, and precise root cause analysis. The experimental results highlight the practical utility of our approach for maintaining service reliability and minimizing downtime in production environments. Our robustness analysis demonstrated that the framework maintained practical effectiveness even when the service dependency information was imperfect. The causal inference module showed remarkable resilience to incomplete (up to 30% missing edges), noisy (up to 15% false edges), and outdated (up to one week old) dependency information. This robustness was achieved through the integration of data-driven causal discovery with prior structural knowledge, enabling the framework to adapt to real-world deployment scenarios where perfect service documentation may not be available.

For future work, we plan to explore several promising directions: (1) **Online Learning**: Enhancing the framework with incremental learning capabilities to adapt to evolving sensed service behaviors and changing patterns, without requiring complete retraining. (2) **Explainable AI**: Integrating more interpretable models and visualization techniques to provide clearer explanations of anomalies and root causes for system administrators. (3) **Predictive Maintenance**: Extending our framework to forecast potential failures before they occur, transitioning from reactive to proactive maintenance strategies. We believe that these enhancements will further improve the practical applicability of our framework in real-world cloud environments and contribute to more reliable and resilient microservice architectures.

## Figures and Tables

**Table 1:** Comparison of evaluation datasets used for generalization analysis.

Characteristic	MultiDimension	AIOps-2020	MicroSim
Number of services	21	26	10/30/50
Metrics per service	19	15	12
Total time series	399	390	120/360/600
Monitoring period	2 weeks	3 weeks	4 weeks
Sampling interval	1 min	2 min	1 min
Anomaly cases	58	72	150
Anomaly types	4 types	5 types	4 types
System domain	General	E-commerce	Simulated
Data source	Real production	Real production	Synthetic

**Table 2:** Anomaly detection performance comparison with 95% confidence intervals (five runs). The bold indicates the best-performing method.

Method	Precision	Recall	F1-Score	AUC-ROC	Delay (s)
LSTM-VAE	0.827±0.015	0.791±0.018	0.809±0.012	0.882±0.009	186±12
MSCRED	0.856±0.013	0.814±0.016	0.834±0.011	0.903±0.008	152±9
OmniAnomaly	0.872±0.011	0.845±0.014	0.858±0.010	0.921±0.007	134±8
InterFusion	0.881±0.010	0.862±0.013	0.871±0.009	0.932±0.006	127±7
TranAD	0.889±0.012	0.870±0.015	0.879±0.010	0.936±0.008	118±6
TCN-only (ours)	0.902±0.009	0.873±0.012	0.887±0.008	0.941±0.005	104±5
VAE-only (ours)	0.891±0.011	0.865±0.014	0.878±0.009	0.933±0.007	122±6
No-Contrastive (ours)	0.917±0.008	0.893±0.010	0.905±0.007	0.952±0.004	95±4
Our Framework	0.938±0.006	0.927±0.008	0.933±0.005	0.965±0.003	78±3

**Table 3:** Fault localization performance comparison with 95% confidence intervals (five runs). The bold indicates the best-performing method.

Method	Precision	Recall	F1-Score	Top-3 Acc.	Mean Rank
MicroCause	0.683±0.019	0.712±0.021	0.697±0.016	0.776±0.018	3.45±0.24
CloudRanger	0.715±0.017	0.742±0.019	0.728±0.014	0.810±0.016	3.21±0.22
MicroRCA	0.747±0.015	0.763±0.018	0.755±0.013	0.845±0.015	2.83±0.19
MIDAS	0.762±0.014	0.781±0.016	0.771±0.012	0.862±0.013	2.56±0.17
CausalRCA	0.791±0.012	0.812±0.014	0.801±0.011	0.879±0.012	2.31±0.15
No-Causal (ours)	0.804±0.011	0.823±0.013	0.813±0.010	0.897±0.010	2.15±0.13
Our Framework	0.876±0.008	0.865±0.009	0.871±0.007	0.931±0.008	1.73±0.09

**Table 4:** Ablation study results with 95% confidence intervals (five runs).

Configuration	Detection F1	Detection Delay	Local. F1	Mean Rank
Full Framework	0.933±0.005	78±3 s	0.871±0.007	1.73±0.09
TCN-only	0.887±0.008	104±5 s	0.815±0.012	2.24±0.15
VAE-only	0.878±0.009	122±6 s	0.807±0.013	2.38±0.17
No-Contrastive	0.905±0.007	95±4 s	0.843±0.010	1.96±0.12
No-Causal	0.927±0.006	82±3 s	0.813±0.010	2.15±0.13
No-Adaptive Threshold	0.914±0.007	91±4 s	0.863±0.008	1.85±0.11
No-EVT	0.922±0.006	85±3 s	0.868±0.008	1.78±0.10

**Table 5:** Sensitivity analysis for sliding window length. The bold indicates the best results.

Window Length	Detection F1	Detection Delay (s)	Localization F1	Training Time (h)	Memory (GB)
10	0.887±0.012	95±6	0.823±0.015	7.23	3.84
20	0.914±0.008	84±4	0.851±0.011	8.12	4.21
30	0.933±0.005	78±3	0.871±0.007	9.34	4.92
40	0.931±0.006	79±4	0.869±0.008	11.47	6.15
50	0.927±0.007	82±5	0.865±0.010	14.28	7.83

**Table 6:** Sensitivity analysis for TCN dilation factors. The bold indicates the best results.

Dilation Factors	Detection F1	Detection Delay (s)	Localization F1	Receptive Field	Params (M)
[1]	0.901±0.009	89±5	0.834±0.012	3	2.1
[1,2]	0.918±0.007	82±4	0.856±0.009	7	4.2
[1,2,4]	0.933±0.005	78±3	0.871±0.007	15	6.3
[1,2,4,8]	0.935±0.005	77±3	0.873±0.006	31	8.4
[2,4,8]	0.925±0.006	81±4	0.863±0.008	28	6.3

**Table 7:** Sensitivity analysis for VAE latent dimensions. The bold indicates the best results.

Latent Dim	Detection F1	Detection Delay (s)	Localization F1	Reconstruction Loss	KL Divergence
8	0.908±0.010	88±5	0.841±0.013	0.0847±0.003	2.31±0.12
16	0.933±0.005	78±3	0.871±0.007	0.0634±0.002	3.47±0.08
32	0.929±0.006	80±4	0.868±0.008	0.0612±0.002	4.83±0.11
64	0.924±0.007	83±4	0.862±0.009	0.0598±0.003	6.24±0.15
128	0.917±0.008	87±5	0.854±0.011	0.0591±0.003	7.91±0.18

**Table 8:** Sensitivity analysis for contrastive learning temperature τ. The bold indicates the best results.

Temperature τ	Detection F1	Detection Delay (s)	Localization F1	Contrastive Loss	Training Stability
0.01	0.892±0.018	94±8	0.831±0.019	1.847±0.045	Unstable
0.05	0.921±0.009	84±5	0.859±0.012	1.234±0.023	Stable
0.07	0.933±0.005	78±3	0.871±0.007	0.987±0.018	Stable
0.1	0.928±0.006	81±4	0.866±0.008	0.856±0.016	Stable
0.2	0.916±0.008	86±5	0.852±0.011	0.634±0.021	Stable
0.5	0.903±0.010	91±6	0.838±0.013	0.421±0.019	Stable

**Table 9:** Computational efficiency comparison.

Method	Training Time (h)	Inference Time (ms)	Memory (GB)
LSTM-VAE	5.23	28.4	3.86
MSCRED	6.78	34.2	4.12
OmniAnomaly	7.42	32.1	4.35
InterFusion	8.15	37.6	4.67
TranAD	6.92	25.8	3.98
MicroCause	–	45.3	2.76
CloudRanger	–	38.7	2.45
MicroRCA	3.56	42.1	3.12
MIDAS	4.87	40.5	3.65
CausalRCA	5.12	43.8	3.42
**Our Framework**	9.34	46.2	4.92

**Table 10:** Impact of incomplete service dependency information on fault localization performance.

Missing Edges	Localization F1	Localization Precision	Localization Recall	Top-3 Accuracy	Mean Rank
0% (Complete)	0.871±0.007	0.876±0.008	0.865±0.009	0.931±0.008	1.73±0.09
10%	0.847±0.009	0.854±0.010	0.841±0.011	0.914±0.011	1.89±0.12
20%	0.821±0.011	0.829±0.012	0.814±0.013	0.896±0.013	2.07±0.15
30%	0.793±0.013	0.802±0.014	0.785±0.015	0.876±0.016	2.28±0.18
40%	0.762±0.015	0.773±0.017	0.752±0.018	0.854±0.019	2.53±0.22
50%	0.728±0.018	0.741±0.020	0.716±0.021	0.829±0.022	2.84±0.26

**Table 11:** Impact of false dependencies (noise) on fault localization performance.

False Edges	Localization F1	Localization Precision	Localization Recall	Top-3 Accuracy	Mean Rank
0% (Clean)	0.871±0.007	0.876±0.008	0.865±0.009	0.931±0.008	1.73±0.09
5%	0.863±0.008	0.869±0.009	0.858±0.010	0.924±0.010	1.79±0.11
10%	0.854±0.009	0.861±0.011	0.848±0.012	0.917±0.012	1.86±0.13
15%	0.842±0.011	0.850±0.013	0.835±0.014	0.908±0.014	1.95±0.16
20%	0.827±0.013	0.836±0.015	0.819±0.016	0.897±0.017	2.08±0.19
25%	0.809±0.015	0.819±0.018	0.800±0.018	0.884±0.020	2.24±0.23

**Table 12:** Impact of outdated service dependency information on fault localization performance.

Dependency Age	Localization F1	Localization Precision	Localization Recall	Top-3 Accuracy	Mean Rank
Current (0 days)	0.871±0.007	0.876±0.008	0.865±0.009	0.931±0.008	1.73±0.09
1 day	0.859±0.008	0.865±0.009	0.854±0.010	0.921±0.010	1.81±0.11
3 days	0.843±0.010	0.851±0.012	0.836±0.013	0.908±0.013	1.94±0.14
7 days	0.821±0.012	0.831±0.014	0.812±0.015	0.891±0.016	2.12±0.18
14 days	0.796±0.014	0.808±0.017	0.785±0.018	0.872±0.019	2.35±0.22

**Table 13:** Performance under combined dependency graph degradation scenarios.

Scenario	Missing	Noise	Age	Localization F1
Perfect Graph	0%	0%	0 days	0.871±0.007
Mild Degradation	10%	5%	1 day	0.834±0.011
Moderate Degradation	20%	10%	3 days	0.796±0.015
Severe Degradation	30%	15%	7 days	0.752±0.019
Extreme Degradation	40%	20%	14 days	0.703±0.023

Note: Best baseline (CausalRCA) achieved 0.623 ± 0.028 under moderate degradation.

**Table 14:** Cross-dataset generalization performance comparison.

Method	Transfer Type	AIOps-2020		MicroSim-30	
		Detection F1	Local. F1	Detection F1	Local. F1
LSTM-VAE	Same-dataset	0.798±0.015	0.734±0.019	0.812±0.012	0.748±0.016
	Direct transfer	0.723±0.021	0.651±0.025	0.736±0.018	0.672±0.021
	Fine-tuned	0.776±0.017	0.708±0.021	0.789±0.014	0.721±0.018
OmniAnomaly	Same-dataset	0.823±0.013	0.761±0.017	0.845±0.011	0.779±0.014
	Direct transfer	0.751±0.019	0.684±0.023	0.768±0.016	0.701±0.019
	Fine-tuned	0.801±0.015	0.738±0.019	0.824±0.012	0.756±0.016
CausalRCA	Same-dataset	0.786±0.016	0.758±0.018	0.801±0.013	0.772±0.015
	Direct transfer	0.698±0.022	0.631±0.026	0.721±0.019	0.654±0.022
	Fine-tuned	0.754±0.018	0.721±0.021	0.778±0.015	0.739±0.018
	Same-dataset	0.918±0.008	0.847±0.011	0.925±0.007	0.861±0.009
**Our Framework**	Direct transfer	0.887±0.012	0.823±0.015	0.901±0.010	0.834±0.013
	Fine-tuned	0.921±0.009	0.854±0.012	0.928±0.008	0.867±0.010

**Table 15:** Scalability analysis on synthetic MicroSim dataset with varying system sizes.

Services	Detection F1	Detection Delay (s)	Localization F1	Training Time (h)	Inference Time (ms)
10	0.941±0.006	71±3	0.883±0.008	4.23	28.4
30	0.925±0.007	78±4	0.861±0.009	9.34	46.2
50	0.912±0.008	84±5	0.847±0.011	16.78	72.6

**Table 16:** Component contribution to transfer learning performance (AIOps-2020 dataset).

Configuration	Detection F1	Localization F1
Full Framework (Direct)	0.887±0.012	0.823±0.015
TCN-only (Direct)	0.834±0.016	0.761±0.019
VAE-only (Direct)	0.821±0.017	0.748±0.021
No-Contrastive (Direct)	0.859±0.014	0.784±0.017
No-Causal (Direct)	0.884±0.013	0.731±0.020
Full Framework (Fine-tuned)	0.921±0.009	0.854±0.012
No-Contrastive (Fine-tuned)	0.903±0.011	0.837±0.014

**Table 17:** Impact of missing value imputation strategies on framework performance.

Strategy	5% Missing		10% Missing		15% Missing		20% Missing	
	Det. F1	Loc. F1	Det. F1	Loc. F1	Det. F1	Loc. F1	Det. F1	Loc. F1
No Imputation	0.847±0.018	0.781±0.023	0.792±0.025	0.718±0.031	0.731±0.032	0.649±0.038	0.664±0.041	0.572±0.047
Forward Fill	0.931±0.006	0.868±0.008	0.928±0.007	0.864±0.009	0.923±0.008	0.858±0.011	0.915±0.010	0.849±0.013
Backward Fill	0.926±0.007	0.861±0.009	0.919±0.009	0.852±0.012	0.908±0.011	0.840±0.014	0.893±0.014	0.824±0.017
Linear Interp.	0.928±0.007	0.865±0.009	0.921±0.008	0.856±0.011	0.909±0.011	0.843±0.014	0.891±0.014	0.825±0.017
Mean Imputation	0.889±0.013	0.816±0.016	0.871±0.015	0.794±0.019	0.847±0.018	0.768±0.022	0.819±0.021	0.738±0.026
Median Imputation	0.893±0.012	0.821±0.015	0.876±0.014	0.801±0.018	0.854±0.017	0.776±0.021	0.827±0.020	0.747±0.025
KNN Imputation	0.914±0.009	0.847±0.012	0.906±0.011	0.836±0.014	0.894±0.013	0.821±0.016	0.878±0.016	0.802±0.019

**Table 18:** Impact of denoising strategies on framework performance under different noise levels.

Strategy	SNR: 40 dB		SNR: 30 dB		SNR: 20 dB		SNR: 10 dB	
	Det. F1	Loc. F1	Det. F1	Loc. F1	Det. F1	Loc. F1	Det. F1	Loc. F1
No Filtering	0.928±0.007	0.865±0.009	0.908±0.011	0.841±0.014	0.876±0.015	0.803±0.019	0.821±0.021	0.742±0.026
Gaussian Filter	0.932±0.006	0.870±0.008	0.929±0.007	0.866±0.009	0.924±0.008	0.859±0.011	0.913±0.010	0.847±0.013
Moving Average	0.925±0.008	0.861±0.010	0.918±0.009	0.852±0.012	0.906±0.011	0.838±0.014	0.887±0.014	0.813±0.018
Savitzky–Golay	0.927±0.007	0.863±0.009	0.921±0.008	0.855±0.011	0.911±0.010	0.843±0.013	0.894±0.013	0.822±0.017
Median Filter	0.923±0.008	0.858±0.010	0.914±0.010	0.847±0.013	0.901±0.012	0.831±0.015	0.882±0.015	0.805±0.019
Wavelet Denoising	0.930±0.006	0.867±0.008	0.925±0.007	0.861±0.010	0.916±0.009	0.849±0.012	0.897±0.012	0.826±0.016

**Table 19:** Performance under combined preprocessing scenarios (10% missing + SNR: 20 dB).

Imputation	Denoising	Detection F1	Localization F1	Processing Time (ms)
Forward Fill	Gaussian Filter	0.918±0.009	0.863±0.012	12.4±0.8
Forward Fill	Moving Average	0.908±0.011	0.851±0.014	15.7±1.2
Forward Fill	Savitzky–Golay	0.912±0.010	0.856±0.013	18.3±1.5
Linear Interp.	Gaussian Filter	0.913±0.010	0.858±0.013	16.9±1.3
KNN	Gaussian Filter	0.905±0.012	0.847±0.015	34.2±2.8
KNN	Wavelet Denoising	0.901±0.013	0.843±0.016	41.6±3.4
Mean	Gaussian Filter	0.867±0.016	0.798±0.020	9.8±0.6
No Imputation	No Filtering	0.774±0.023	0.712±0.028	3.2±0.3

**Table 20:** Computational overhead analysis of different preprocessing strategies.

Strategy	Processing Time (ms)	Memory Overhead (%)	CPU Usage (%)	Scalability
**Imputation Methods:**				
Forward Fill	3.2±0.3	1.2±0.1	2.1±0.2	Excellent
Backward Fill	3.4±0.3	1.3±0.1	2.3±0.2	Excellent
Linear Interpolation	8.7±0.7	2.1±0.2	3.8±0.3	Good
Mean Imputation	5.1±0.4	1.8±0.2	2.9±0.3	Good
KNN Imputation	28.4±2.3	5.7±0.5	8.2±0.7	Moderate
**Denoising Methods:**				
Gaussian Filter	9.2±0.6	0.8±0.1	1.7±0.2	Excellent
Moving Average	12.5±0.9	1.1±0.1	2.3±0.2	Excellent
Savitzky–Golay	15.1±1.2	1.4±0.1	2.8±0.3	Good
Median Filter	11.8±0.8	1.0±0.1	2.1±0.2	Good
Wavelet Denoising	32.7±2.6	3.2±0.3	5.4±0.5	Moderate

**Table 21:** Performance under dynamic service addition/removal scenarios.

Change Rate	Detection Performance		Localization Performance		Adaptation Time	
	**F1-Score**	Delay (s)	F1-Score	Mean Rank	Detection (min)	Localization (min)
Static Baseline	0.933±0.005	78±3	0.871±0.007	1.73±0.09	N/A	N/A
Low (1/day)	0.921±0.008	83±5	0.854±0.011	1.89±0.14	67±12	94±18
Moderate (3/day)	0.912±0.010	87±6	0.841±0.013	2.06±0.19	78±15	112±24
High (5/day)	0.901±0.012	92±8	0.824±0.016	2.31±0.26	89±21	134±31

Note: Performance measured during stable periods between changes.

**Table 22:** Impact of dependency rearrangement on framework performance.

Rewiring %	6-h Interval		12-h Interval		24-h Interval	
	Det. F1	Loc. F1	Det. F1	Loc. F1	Det. F1	Loc. F1
0% (Static)	0.933±0.005	0.871±0.007	0.933±0.005	0.871±0.007	0.933±0.005	0.871±0.007
10%	0.918±0.009	0.847±0.012	0.925±0.007	0.859±0.010	0.929±0.006	0.865±0.009
20%	0.897±0.013	0.816±0.017	0.912±0.010	0.841±0.014	0.921±0.008	0.854±0.011
30%	0.871±0.017	0.781±0.022	0.894±0.014	0.818±0.018	0.908±0.011	0.838±0.015

**Table 23:** Performance comparison between gradual and abrupt topology changes.

Change Type	During Transition		Post-Adaptation		Recovery Time	
	Det. F1	Loc. F1	Det. F1	Loc. F1	Detection	Localization
**Service Addition:**						
Gradual (6 h)	0.908±0.012	0.831±0.016	0.927±0.008	0.861±0.011	89±18 min	126±28 min
Abrupt	0.847±0.019	0.763±0.024	0.921±0.009	0.854±0.013	142±32 min	198±45 min
**Service Removal:**						
Gradual (6 h)	0.912±0.011	0.841±0.014	0.929±0.007	0.864±0.010	52±12 min	73±16 min
Abrupt	0.863±0.017	0.784±0.021	0.924±0.008	0.858±0.012	78±19 min	115±26 min
**Dependency Rewiring:**						
Gradual (6 h)	0.901±0.013	0.823±0.017	0.922±0.009	0.856±0.012	134±24 min	187±38 min
Abrupt	0.834±0.021	0.741±0.027	0.915±0.011	0.847±0.015	213±48 min	298±67 min

**Table 24:** Effectiveness of different topology change detection mechanisms.

Detection Mechanism	Detection Rate	False Positive	Trigger Delay	Adaptation Trigger	Effectiveness
Correlation Pattern Change	0.847±0.023	0.094±0.012	23±8 min	High sensitivity	Good
New Metric Signatures	0.923±0.015	0.067±0.009	12±4 min	Service addition	Excellent
Prediction Error Spike	0.891±0.018	0.123±0.015	18±6 min	All change types	Good
Causal Graph Likelihood	0.876±0.021	0.058±0.007	31±12 min	Dependency changes	Excellent
Combined Indicators	0.934±0.011	0.045±0.006	15±5 min	All types	Outstanding

**Table 25:** Performance comparison during topology transitions: adaptive vs. static approaches.

Approach	During Transition		Retraining Required		Downtime	
	Det. F1	Loc. F1	Time (hours)	Data Required	Detection	Localization
**Our Adaptive Framework**	0.891 ± 0.015	0.816 ± 0.019	0	None	0 min	0 min
LSTM-VAE (Static)	0.623 ± 0.034	0.487 ± 0.041	8–12	Full dataset	480–720 min	480–720 min
OmniAnomaly (Static)	0.651 ± 0.031	0.509 ± 0.038	6–10	Full dataset	360–600 min	360–600 min
CausalRCA (Static)	0.587 ± 0.037	0.441 ± 0.044	10–15	Full dataset	600–900 min	600–900 min

Note: Static methods require complete retraining after topology changes.

## Data Availability

The original contributions presented in this study are included in the article. Further inquiries can be directed to the corresponding author.
